# Low premorbid IQ may exacerbate the cognitive effects of apolipoprotein ε4 (APOE ε4): a multi-ethnic cross-sectional study from HABS-HD

**DOI:** 10.3389/fneur.2025.1627525

**Published:** 2025-07-08

**Authors:** Lubnaa Badriyyah Abdullah, Zhengyang Zhou, Ney Alex Alliey, Robert Barber, James Hall, Sid O’Bryant

**Affiliations:** ^1^Department of Family Medicine, University of North Texas Health Science Center (UNTHSC), Fort Worth, TX, United States; ^2^Department of Population and Community Health, UNTHSC, Fort Worth, TX, United States; ^3^Department of Psychological Sciences, University of Texas Rio Grande Valley, Edinburg, TX, United States

**Keywords:** Alzheimer’s disease (AD), apolipoprotein (APOE), cognition, premorbid IQ, intellectual disability, biomarkers

## Abstract

**Introduction:**

Apolipoprotein allele 4 (APOE ε4) is associated with lower IQ scores during childhood and adolescence, but the influence of APOE ε4 and low IQ on late-life cognition is unknown. This study examines the association between APOE ε4 and cognitive outcomes based on premorbid intellectual ability (pIQ) and ethnic background.

**Methods:**

Participants were drawn from the Health & Aging Brain Study–Health Disparities (HABS-HD), categorized by low (*z* ≤ −2.00) or average (*z* = 0.00 ± 1.00) pIQ based on word reading scores. Statistical analyses were conducted to evaluate whether APOE ε4 was associated with the cognitive domains of episodic memory, executive functioning, processing speed, and language by pIQ and ethnicity.

**Results:**

APOE ε4 was associated with worse cognitive performance across domains. In the overall sample analysis, the deleterious effect of ε4 on processing speed and executive functioning was stronger among those with low pIQ. In stratified analysis, the negative impact of APOE ε4 was stronger among non-Hispanic White individuals with low pIQ for episodic memory and Hispanic individuals with low pIQ for processing speed.

**Discussion:**

The influence of APOE genotype on cognitive outcomes is moderated by ethnicity and premorbid IQ, positioning low pIQ, a proxy for intellectual disability (ID), as a population more vulnerable to the negative effects of APOE ε4 in older adulthood.

**Conclusion:**

The effect of Alzheimer’s disease (AD) risk genes on cognitive performance may not mirror what is observed in AD-Down syndrome, highlighting the urgent need to expand AD research to reach more representative populations with I/DD.

## Introduction

The increased risk for dementia in older age for people living with intellectual disability (ID) without Down syndrome (DS) is not well understood; however, there may be multiple cognitive, biological, and genetic pathways that increase the risk of dementia in this group (1). Genetic risks may be moderated by the etiology of ID and the aggregate impacts of Alzheimer’s Disease (AD) risk genes, such as apolipoprotein (APOE), on dementia outcomes. APOE is the body’s main cholesterol transporter, playing additional roles in synaptic plasticity and cell signaling. APOE has three alleles, ε2, ε3, and ε4, all of which have a differential impact on AD risk (2). A ε4/ε4 genotyping carries an established dose-dependent risk for late-onset AD^1^ and also plays a role in non-pathological cognitive aging, with associations with cognitive ability and greater impairment in memory and processing speed with age (3). Among those with DS, a common form of ID, APOE allele ε4 (APOE ε4) is associated with increased mortality and earlier age of onset of AD (4–6). This suggests that APOE ε4 genotyping compounds upon this causal risk for AD in DS associated with the trisomy of the amyloid precursor protein (APP). DS is genetically and biologically distinct from other forms of ID and carries a specific AD risk profile, precluding generalizations to those with non-DS ID. Therefore, to begin addressing this literature gap, the present study explores the relationship between APOE genotyping and cognitive performance in an ethnically diverse group of individuals with low premorbid IQ (pIQ), equivalent to ID without DS.

The ε4/ε4 variant combinations carry the greatest risk of AD; however, the ancestry around the APOE ε4 gene determines risk, not the gene itself. Individuals of African ancestry generally have a higher prevalence of APOE ε4 compared to those of European and Asian descent, yet experience less risk of AD associated with this gene when compared to European ε4 carriers (7). European carriers with two copies of APOE ε4 have at least a 10-fold risk of AD compared to those with other variants (8). Among Asian APOE ε4 carriers, there is a higher risk for AD than European carriers (9). Furthermore, the effects of APOE ε4 on cognition in Hispanic ethnic groups are weaker and inconsistent (9). An individual who receives APOE ε4 from an African ancestor will therefore have an African risk for AD associated with the gene, and so on. The exact mechanisms underlying this differential risk mediated by ethnicity are not well understood. Admixture and environmental and lifestyle factors, nevertheless, modulate the effect of APOE ε4 (10–12). Some researchers have suggested that reactive astrocytes in the region of DNA surrounding APOE ε4 have important information about the production of APOE, citing 40% more APOE transcripts at autopsy from European carriers and increased A1 reactive astrocytes in these transcripts (10).

There may be early associated impacts of APOE ε4 on intellectual ability that impact later permeability of the APOE ε4 AD risk gene, warranting further inquiry. APOE ε4 is associated with lower verbal and full-scale IQ scores during childhood and adolescence (13). Moreover, previous research has shown that there are differences in brain volume, neuropsychological performance, and fractional anisotropy by APOE ε4 genotypes among children and young adults, suggesting that structural brain differences in carriers’ brain volumes appear in infancy and continue into adulthood (14). Lower density of gray and white matter and lower hippocampal, frontal, and temporal lobe volumes have been noted in infant APOE ε4 carriers aged 2 to 25 months. A 20-year longitudinal cohort study (Whitehall II Study) on the association of APOE ε4 with cognitive function over the adult life course found that ε4 homozygotes had poorer global cognitive function starting from 65 years; ε4 heterozygotes had better cognitive scores between the ages of 45–55 years, then no difference until poorer cognitive scores from 75 years and onwards (15, 16). This suggests a midlife benefit of APOE ε4 that is not attenuated in old age, suggesting critical periods during neurodevelopment and neurodegeneration that may impact aging in APOE ε4 carriers (16).

Partnering with community-dwelling research participants from the multi-ethnic community-dwelling cohort, the Health and Aging Brain Study-Health Disparities (HABS-HD), we use word reading, an accepted proxy (17) for one’s premorbid intellectual ability to stratify participants into low and average (avg.) pIQ groups. This study aims to (1) identify characteristic differences in APOE gene distribution among those with low and avg. pIQ, (2) report the associations of APOE ε4 on cognitive outcomes among those with low and avg. pIQ, and (3) examine the effects of ethnicity on gene–cognition associations.

## Methods

HABS-HD is an ongoing, longitudinal, community-based study of health disparities in brain health in underrepresented populations, with specific recruitment for Hispanic, non-Hispanic White (NHW), and non-Hispanic Black (NHB) ethnicity. Data Release 6 was processed for this study, previously released in November 2024. All study procedures are completed at one time-point using baseline cross-sectional data. Participants for the current study were selected based on the American National Reading Test (AMNART) (18) and Word Accentuation Test (WAT) (see text footnote 1) for Spanish speakers. Individuals with low or average word reading were grouped by the following parameters: low-premorbid IQ (low pIQ; *z*-score < −2.00) and average premorbid IQ (avg pIQ; *z*-score = 0.00 ± 1.00). These criteria were selected based on (1) the normal bell curve and associated classification of stanines and (2) the definition of ID as defined by the Diagnostic and Statistical Manual of Mental Disorders, fifth Edition, to be an IQ at least 2 SD below the mean (19).

### Word reading protocol

The WAT (20) is used to assess Spanish speakers’ ability to place stress or accent on words correctly. The WAT assesses Spanish speakers’ premorbid IQ by reading the correct pronunciation of 30 low-frequency Spanish words whose accents have been removed. The person is asked to read each word aloud. The test administrator records the number of words the participant pronounces correctly. The overall score is used to estimate the participant’s premorbid IQ. AMNART (18) is a neuropsychological assessment used to estimate premorbid intellectual functioning by evaluating a person’s ability to read aloud a list of phonetically irregular words. The test consists of 50 words that are irregular in their spelling-to-sound correspondence. Participants are asked to read these words aloud, with the primary goal of assessing their ability to correctly pronounce these irregular words. The test administrator records the number of words the participant pronounces correctly. The overall score is used to estimate the participant’s premorbid IQ. Z-scores are calculated using normative references from the HABS-HD cohort classified by ethno-racial group, education (i.e., 0–7 years, 8–12 years, and 13 + years), primary language (English or Spanish), and age (median split <=65 and > = 66).

### Cognitive assessment

The HABS-HD protocol includes the following cognitive assessments: Mini Mental Status Exam (MMSE) (21), Wechsler Memory Scale-Third Edition (WMS-III) Digit Span and Logical Memory (21), Digit Symbol Substitution, Trail Making Test Parts A and B (21), Spanish–English Verbal Learning Test (SEVLT) (22), Animal Naming (semantic fluency) (21), F-A-S (phonemic fluency) (21), the American National Adult Reading Test (English speakers) (21), and Word Accentuation Test (Spanish speakers) (20). *Z*-scores are calculated using ethno-racial specific normative references from the HABS-HD cohort classified by education (i.e., 0–7 years, 8–12 years, and 13 + years), primary language (English or Spanish), and age (median split <=65 and > = 66) (18). An informant interview is also conducted by clinicians with expertise in dementia to evaluate functional declines for completion of the Clinical Dementia Rating (CDR) Scale (21, 23).

### Cognitive domains

Standardized z-scores from the neuropsychological tests were used in the analysis, resulting in 10 total outcomes. Z-scores were then translated into cognitive domains by taking the averages of the *z*-scores for each participant. Table 1 shows the individual test scores that were combined to create each domain.

**Table 1:** Neuropsychiatric testing individual tests by domain and cognitive function.

Neuropsychiatric test	Episodic memory	Executive functioning	processing speed	Language
SEVLT	X			
SEVLT-DR	X			
WMS-III LM1	X			
WMS-III LM2	X			
WMS-III DSF		X		
WMS-III DSB		X		
TMT A			X	
TMT B			X	
Animal Naming				X
F-A-S Verbal Fluency				X

### APOE genotyping

Blood samples were analyzed at the University of North Texas Health Science Center’s Institute for Translational Research (ITR) laboratory by the biomarker core. Collection of fasting blood samples was processed within 120 min of draw, clotted in a vertical position at room temperature before centrifugation, and inverted 5–10 times. Samples were stored in aliquots no larger than 0.5 mL at −80° (24). DNA was extracted from peripheral blood buffy coat samples to assay and genotype for apolipoprotein ε4. Individuals with at least one copy of APOE ε4 were considered carriers.

### Recruitment, protocol, and inclusion

All procedures are conducted under IRB-approved protocols. Using a community-based participatory research approach, participants were recruited from the greater Dallas-Fort Worth community (25). Participants (or their legally authorized representatives [LARs]) provided written informed consent. The HABS-HD protocol includes interviews, functional exams, blood draws for clinical labs and biobanking, neuropsychological testing, and 3 T Magnetic Resonance Imaging (MRI) scans of the brain. Amyloid and tau Positron Emission Tomography (PET) scans are ongoing for the full cohort. The study protocol can be conducted in Spanish or English. Data from the study are accessible to the scientific community through the UNTHSC Institute for Translational Research website. Inclusion in HABS-HD requires (1) willingness to provide blood samples, (2) the ability to undergo neuroimaging studies, (3) age 30 and above, and (4) fluency in English or Spanish. Exclusion criteria include: (1) type 1 diabetes, (2) presence of active infection, (3) current/recent (12 month) cancer (other than skin cancer), (4) current severe mental illness that could impact cognition (other than depression), (5) recent (12 months) traumatic brain injury with loss of consciousness, (6) current/recent alcohol/substance abuse, and (7) active severe medical condition that could impact cognition (e.g., end-stage renal failure, chronic heart failure, and chronic obstructive pulmonary disease).

## Statistical analysis

Analyses were conducted in R (version 4.3.2; R Foundation for Statistical Computing, Vienna, Austria); IBM SPSS Statistics (version 29; IBM Corp., Armonk, NY, USA). Multiple linear regression models were estimated to evaluate the associations between APOE ε4 and each of the cognitive domains. Cognitive domain scores were calculated by taking the averages of the *z*-scores of domain-relevant cognitive measures that were available in the HABS-HD database (Table 1). To examine if the associations between APOE ε4 and cognitive domains differ between participants in low and average pIQ groups, the interaction terms between pIQ group and APOE ε4 were included in the regression analysis. To understand the impact of ethnicity on the above associations, an overall analysis was conducted on the entire sample (i.e., NHB, Hispanic, and NHW individuals combined), and three stratified analyses were conducted on each of the NHB, Hispanic, and NHW racial/ethnic groups. Regression models controlled for age, sex, and education as covariates in all analyses, with ethnic group included as an additional covariate in the overall analysis. Statistical significance was set at *p* < 0.05.

## Results

### Demographic characteristics

Data were analyzed from n = 528 NHB, *n* = 785 Hispanic, and *n* = 898 NHW individuals in the average premorbid IQ group (77.6% of the group are English speakers) and *n* = 35 NHB, *n* = 43 Hispanic, and *n* = 31 NHW individuals in the low premorbid IQ group (86.1% are English speakers). Table 2 shows the characteristics of the sample by pIQ, with summaries of the age distribution, APOE genotyping, and female sex distribution. APOE ε4 carriership is also reported.

**Table 2:** Descriptive characteristics of HABS:HD study participants separated by pIQ group.

Low pIQ	Total sample	NHB (*n* = 35)^1^	Hispanic (*n* = 43)	NHW (*n* = 31)	*p*-value^2^
Age	66.26 (9.40)	63.66 (8.12)	65.07 (7.98)	70.84 (11.06)	**0.014**
Education	14.23(2.84)	15.00 (2.36)	12.95 (3.05)	15.16 (2.38)	**0.002**
% Female	59 (54%)	18 (51%)	28 (65%)	13 (42%)	0.13
APOE ε4					0.3
ε4 non-carriers	82 (75%)	23 (66%)	37 (86%)	22 (71%)	
ε4	21 (19%)	9 (26%)	5 (12%)	7 (23%)	
ε4/ε4	6 (5.5%)	3 (8.6%)	1 (2.3%)	2 (6.5%)	

Avg pIQ	Total sample	NHB (*n* = 582)	Hispanic (*n* = 785)	NHW (*n* = 898)	*p*-value
Age	65.20 (8.62)	63.08 (7.81)	62.95 (7.97)	68.53 (8.61)	**<0.001**
Education	14.14 (3.50)	14.91 (2.56)	11.91 (3.94)	15.61 (2.47)	**<0.001**
% Female	1,435 (63%)	396 (68%)	511 (65%)	528 (59%)	**<0.001**
APOE ε4					**<0.001**
ε4 non-carriers	1,595 (70%)	345 (59%)	625 (80%)	625 (70%)	
ε4	607 (27%)	202 (35%)	154 (20%)	251 (28%)	
ε4/ε4	63 (2.8%)	35 (6.0%)	6 (0.8%)	22 (2.4%)	

^1^Mean (SD); *n* (%).

^2^Kruskal–Wallis rank-sum test; Fisher’s exact test; Pearson’s chi-squared test.

*p* < 0.05. Significant *p* values are reflected in bold.

### APOE ε4 and cognitive outcomes

Table 3 shows the association of APOE ε4 with cognitive outcomes across the entire sample. APOE ε4 was significantly associated with worse performance in episodic memory, processing speed, and language across the entire sample in a dose-dependent manner, but there were no associations between executive functioning and APOE ε4 across the entire sample. Table 4 shows the associations of APOE ε4 and cognitive domains stratified by ethnicity. APOE ε4 was associated with worse episodic memory among NHB participants, regardless of pIQ and NHW participants with low pIQ, whereas worse processing speed and language performance were singularly associated with APOE ε4 in NHB individuals, regardless of pIQ level. Hispanic APOE ε4 carriers with low pIQ had significantly worse performance in processing speed.

**Table 3:** Association of pIQ with cognitive outcomes and APOE ε4 carriership across the total sample.

Cognitive domain	Variable	Estimate	Std. Error	*t*-value	*p*-value
Episodic Memory	APOE ε4	−0.16	0.03	−4.99	**< 0.001**
	pIQ: APOE ε4	−0.24	0.14	−1.69	0.09
Executive Functioning	APOE ε4	−0.04	0.04	−1.15	0.25
	pIQ: APOE ε4	−0.32	0.16	−2.06	**0.04**
Processing Speed	APOE ε4	−0.08	0.03	−2.16	**0.03**
	pIQ: APOE ε4	−0.34	0.15	−2.33	**0.02**
Language	APOE ε4	−0.11	0.05	−2.03	**0.04**
	pIQ: APOE ε4	−0.02	0.22	−0.12	0.91

*p* < 0.05. Significant *p* values are reflected in bold.

**Table 4:** Association of pIQ with cognitive outcomes and APOE ε4 carriership across three ethnic groups.

Cognitive domain	Ethnicity	Variable	Estimate	Std. error	*t*-value	*p*-value
Episodic Memory	NHB	APOE ε4	−0.19	0.06	−3.34	**< 0.001**
		pIQ: APOE ε4	0.23	0.22	1.02	0.31
	Hispanic	APOE ε4	−0.12	0.07	−1.85	0.06
		pIQ: APOE ε4	−0.49	0.29	−1.69	0.09
	NHW	APOE ε4	−0.18	0.05	−3.45	**< 0.001**
		pIQ: APOE ε4	−0.51	0.25	−2.1	**0.04**
Executive Functioning	NHB	APOE ε4	−0.12	0.06	−1.99	**0.05**
		pIQ: APOE ε4	0.1	0.24	0.39	0.70
	Hispanic	APOE ε4	0.06	0.08	0.71	0.48
		pIQ: APOE ε4	−0.67	0.35	−1.92	0.05
	NHW	APOE ε4	−0.05	0.06	−0.89	0.37
		pIQ: APOE ε4	−0.23	0.26	−0.87	0.39
Processing Speed	NHB	APOE ε4	−0.11	0.05	−2.03	**0.04**
		pIQ: APOE ε4	−0.02	0.22	−0.12	0.98
	Hispanic	APOE ε4	−0.02	0.07	−0.34	0.74
		pIQ: APOE ε4	−0.66	0.32	−2.08	**0.04**
	NHW	APOE ε4	−0.08	0.06	−1.45	0.15
		pIQ: APOE ε4	−0.42	0.26	−1.62	0.11
Language	NHB	APOE ε4	−0.12	0.05	−2.14	**0.03**
		pIQ: APOE ε4	0.22	0.21	1.01	0.31
	Hispanic	APOE ε4	−0.01	0.07	−0.15	0.88
		pIQ: APOE ε4	−0.32	0.29	−1.1	0.27
	NHW	APOE ε4	−0.06	0.05	−1.31	0.19
		pIQ: APOE ε4	−0.19	0.23	−0.85	0.4

*p* < 0.05. Significant *p* values are reflected in bold.

### APOE ε4 interaction effects with pIQ status

**Figure 1:**
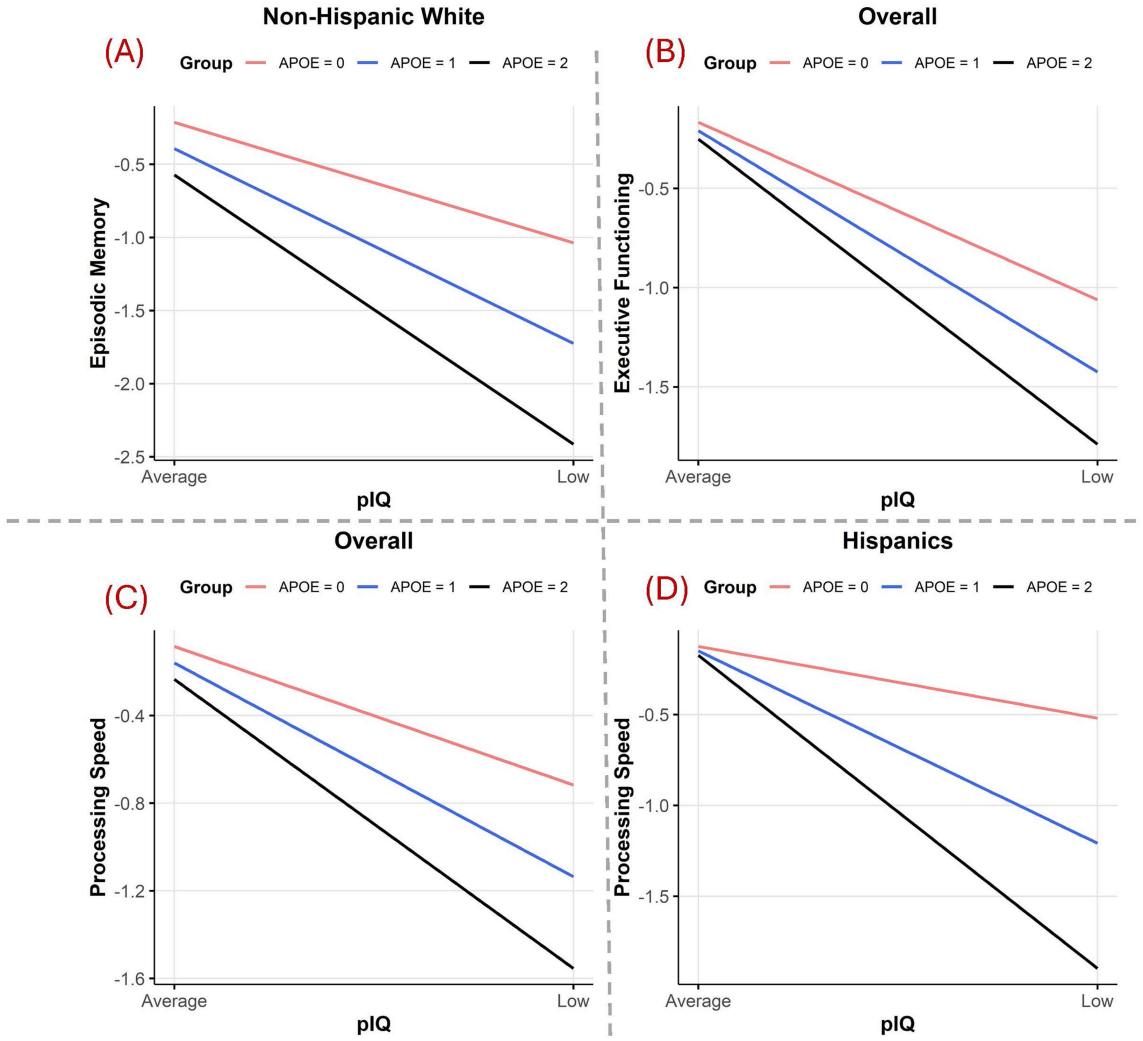
Interaction plots show the associations between pIQ status and cognitive domains across APOE genotypes. **(A)** Interaction between the pIQ status and APOE genotype on episodic memory for the NHW group. **(B)** Interaction between the pIQ status and APOE genotype on executive functioning for the overall population. **(C)** Interaction between the pIQ status and APOE genotype on processing speed for the overall population. **(D)** Interaction between the pIQ status and APOE genotype on processing speed for the Hispanic group.

Table 4 shows the interaction terms (relative strength of associations) between pIQ and APOE ε4 across three ethnic groups. There were no significant interactions between APOE ε4 and pIQ across the language and executive functioning domains. There was a significant interaction (stronger effect) of APOE ε4 on episodic memory for NHW participants with low pIQ (Figure 1A), with no other significant interactions. Across all participants, APOE ε4 had a significantly stronger effect on executive functioning (Figure 1B) and processing speed performance (Figure 1C) for those with low pIQ. This interaction for processing speed was observed only in Hispanic participants in stratified analyses (Figure 1D).

## Discussion

In this multiethnic cross-sectional study, we examined the relationship between APOE ε4 and cognitive outcomes among those with low and avg. premorbid IQ (pIQ). The analysis evaluated the effect of APOE ε4 on each of the four cognitive domains and how that effect might differ across pIQ groups (i.e., interaction terms). Our results reinforce the established role of APOE ε4 in cognitive performance across memory, cognitive processing, and language (26–28). Consistent with the literature, individuals who carry a higher number of ε4 alleles tended to have lower scores in cognitive performance in a dose-dependent manner. Overall, our findings did not reveal a consistent interaction between APOE ε4, low pIQ, and cognitive outcomes. In the overall sample, the negative associations between APOE ε4 carriership and executive functioning and processing speed were stronger among APOE ε4 carriers with low pIQ. In stratified analysis, significant effects emerged only on processing speed among Hispanic participants and episodic memory among NHW participants. Our results highlight that the cognitive impact of APOE ε4 varies by both premorbid IQ and ethnicity, and this variability is not uniform. Our results reflect that ancestry not only modifies the cognitive effects of the APOE ε4, but also there may be potential gene-by-environment interactions that underlie distinct patterns of cognitive vulnerability across ethnic groups of varying intellectual abilities. These preliminary results suggest that the relationship between APOE ε4 and cognitive performance in those with developmentally low intellectual ability does not mirror what is observed in late-onset or autosomal-dominant forms of AD, warranting further scientific inquiry.

Within the processing speed and executive domains, the negative impact of APOE ε4 was significantly more pronounced for those with low pIQ. Processing speed, being linked to white matter integrity, is observed to decline in APOE ε4 carriers due to impaired myelination, axonal injury, and reduced repair mechanisms (29). Our findings suggest that cognitive differences associated with APOE ε4 are not only evident in early life—as previously reported in the literature—but are also potentially magnified later in the life course for those with developmental low IQ (30). The importance of early intervention, prior to mid-life, during childhood or adolescence, therefore, may be a key window to enhance cognitive resiliency in at-risk individuals with low IQ. We have previously established higher levels of plasma total tau in Hispanic individuals with low pIQ, suggesting greater neurodegeneration and neuronal deficiencies in this group (31). Our findings add breadth, suggesting that Hispanic ε4 carriers with low pIQ may be vulnerable to neurodegeneration changes that impact cognitive efficiency, potentially related to lifestyle factors and co-occurring metabolic conditions. Hispanic APOE ε4 carriers with low pIQ may have less cognitive resilience, making early disruption in neural transmission more evident in this domain (32). Dysregulated lipid metabolism is a well-established contributor to AD pathogenesis, with cerebral cholesterol accumulation linked to accelerated synaptic deficiencies, neuroinflammation, white matter abnormalities, Aβ production, and cognitive decline (33, 34). Our findings may reflect the aggregate consequence of metabolic factors, developmentally low IQ, and APOE ε4, on neural efficiency in Hispanic individuals with low pIQ. Through the promotion of neuroinflammatory responses and dysregulated lipid metabolism, multiple mechanistic pathways may contribute to cognitive changes and neurodegeneration. Our findings suggest a more nuanced relationship between APOE ε4 and cognitive processing, which may be influenced by the interplay of cognitive ability, age, ethnicity, lifestyle, and underlying physiological brain changes (35, 36).

Only NHW participants with low pIQ had worse memory scores related to the presence of APOE ε4. This suggests that even among NHW individuals with lower cognitive ability, the broader trend of cognitive decline related to European ancestry around APOE ε4 persists. Nevertheless, future studies should consider all polymorphisms of APOE across an ethnically diverse sample with ID to test this relationship further. Among Hispanic participants, there is no association of APOE ε4 with episodic memory, adding to the breadth of literature that supports an inconsistent relationship regarding APOE ε4 and memory in this group (37). Across the total NHB sample, APOE ε4 was significantly associated with worse episodic memory, language, and processing speed performance. Results replicate prior findings among NHB individuals associating APOE ε4 with worsened cognitive ability (38). However, among NHB people, behavioral factors, such as social activities and stress levels, have independent and additive effects on cognitive status, which may partially impact the current results (38). Disruptions in lipid transport pathways may represent promising therapeutic targets for AD prevention and treatment in NHB Americans, as there also may be additive vascular factors—such as small vessel ischemic disease, hypertension, hypercholesterolemia, diabetes, and stress—more prevalent among NHB individuals that impact the observed relationships (30, 39). Moreover, the interaction of APOE ε4 with cognition in NHB participants may have different roles across the lifespan, and future directions should consider longitudinal designs. Taken together, we present compelling preliminary evidence that highlights the importance of examining genetic risk factors within specific ethnic and cognitive subgroups to better understand the nuanced ways APOE ε4 influences cognitive aging.

## Limitations and conclusion

The cross-sectional nature of the study limits the ability to conclude causal relationships between APOE ε4, cognitive outcomes, and pIQ. Although individuals with low pIQ were relatively evenly distributed across racial and ethnic groups, the subgroup sample sizes remained small, which may restrict the generalizability of the observed effects. Additionally, although pIQ serves as a useful proxy for ID, it does not fully capture the heterogeneity inherent in ID, which encompasses a broad range of cognitive functioning and adaptive behaviors. Although word reading is a common proxy for adult IQ, it may introduce bias, as these measures are less reliable at the extremes of the IQ distribution and may also be influenced by APOE ε4, which has been linked to lower childhood verbal IQ (13, 40). Future research should prioritize cohorts with formally diagnosed intellectual and developmental disabilities, enabling a more precise investigation into the mechanisms by which genetic and environmental risk factors shape trajectories of cognitive aging and AD vulnerability in this underserved population. Importantly, the influence of APOE ε4 on cognition may vary across the lifespan, potentially affecting early neurodevelopmental processes, midlife cognitive maintenance, and late-life neurodegeneration in distinct ways. Longitudinal studies are therefore essential to better characterize how APOE ε4 interacts with both cognitive resilience and cognitive vulnerability over time.

## Data Availability

The datasets presented in this study can be found in online repositories. The names of the repository/repositories and accession number(s) can be found at: https://apps.unthsc.edu/itr/reports.

## Footnotes

^1^ https://www.zotero.org/google-docs/?broken=ZIt5Ir
